# Efficacy and nephrotoxicity of polymyxin B in elderly patients with carbapenem resistant bacterial infection

**DOI:** 10.1186/s12941-023-00647-2

**Published:** 2023-11-15

**Authors:** G. L. Xia, X. Xu, X. B. You, X. Wang, D. D. Feng, S. Lei, R. L. Jiang

**Affiliations:** 1 Department of Intensive Care Unit, The First Affiliated Hospital of Zhejiang Chinese Medical University (Zhejiang Provincial Hospital of Traditional Chinese Medicine), NO.54 Youdian Road, Hangzhou, 310006 China; 2 The First School of Clinical Medicine, Zhejiang Chinese Medical University, Hangzhou, 310053 China; 3 The Third Affiliated Hospital of Zhejiang, Chinese Medical University, Hangzhou, 310009 China

**Keywords:** Polymyxin B, Elderly, Acute kidney injury, Infection, Carbapenem-resistant organism

## Abstract

**Background:**

To study the efficacy and nephrotoxicity of polymyxin B in the treatment of elderly patients with carbapenem-resistant organism (CRO) infection.

**Methods:**

The clinical and microbiological data of patients with CRO-infected sepsis treated with polymyxin B were retrospectively analyzed. The effective rate, bacterial clearance, incidence and recovery rate of acute renal injury (AKI) and prognosis-related indicators in AKI at different stages were compared.

**Results:**

The effective rate of 215 elderly patients with CRO infection treated with polymyxin was 50.7%. The total bacterial clearance rate was 44.2%, the total incidence of AKI was 37.2%, the recovery rate of AKI was 35%, and the incidence range of polymyxin B-related AKI was 10.2–37.2%. Logistic multivariate regression analysis showed that the predictors of AKI in elderly patients were high APACHE II score, long duration of polymyxin, chronic renal insufficiency and ineffective outcome; the ROC curve showed that the cutoff value for predicting AKI was a serum creatinine concentration of 73 mmol/L before polymyxin B use, and the AUC was 0.931.

**Conclusions:**

Rational use of polymyxin B is safe and effective in elderly patients with CRO infection, and its effective outcome can improve the recovery rate of AKI.

## Background

The infection of carbapenem-resistant organisms (CRO) is becoming increasingly serious [1]. It has been reported that CRO easily takes elderly patients as a host when they are in a hospital environment, and the blood flow infection of elderly patients increases 16 times after admission [2]. Polymyxin is the last line of defense in the treatment of patients with CRO infection, but its high incidence of nephrotoxicity is also an indisputable fact [3]. Especially for elderly patients, the pharmacokinetics/pharmacodynamics (PK/PD) of drugs have changed greatly due to their poor renal reserve function, combined with shock and a heavy systemic inflammatory response [4], which may be more prominent risk factors for polymyxin-associated acute renal injury (AKI). This puts clinicians in a dilemma in drug selection for elderly infected patients. Therefore, it is very important to understand the incidence and possible risk factors for polymyxin-associated AKI in elderly infected patients, which is of great significance to optimize the treatment plan and object selection, to reduce the occurrence of renal injury and improve the prognosis of elderly patients.

Polymyxin is a cationic peptide antibiotic that can quickly kill bacteria by destroying the integrity of the cell membrane [5]. It has been widely used to treat gram-negative bacteria. Later, it was replaced by other antibiotics because of its narrow antibacterial spectrum and strong nephrotoxicity. With the emergence of *carbapenem-resistant Enterobacteriaceae (CRE)*, *carbapenem-resistant Acinetobacter baumannii (CRAB)*, *carbapenem-resistant Pseudomonas aeruginosa (CRPA)* and other carbapenem-resistant bacteria worldwide, polymyxin, as an effective antibacterial drug for the treatment of gram-negative bacterial infection, has received attention [6]. Polymyxin B sulfate and polymyxin E sulfate are currently used clinically. Compared with polymyxin E sulfate, polymyxin B shows less PK variability because it is administered in its pharmacologically activity form [7]. Current research shows that it has a good curative effect on nosocomial CRO infection [6]. Clinical PK studies have shown that polymyxin B will not be cleared through the kidney, and its clearance rate has nothing to do with renal function [8]. Therefore, it is speculated that the incidence and severity of AKI associated with polymyxin B treatment are limited. However, many studies have reported that polymyxin B has nephrotoxicity, and the incidence of nephrotoxicity is 24.7–54.9% [9, 10]. There are great differences in the literature, partly due to the different definitions of nephrotoxicity, the wide range of polymyxin B dose regimens and the heterogeneity of patients.

Considering the potential toxicity associated with polymyxin B overdose treatment exposure and the increased risk of treatment failure associated with subdose treatment exposure, the question of “whether renal function is important” in polymyxin B administration is a key issue worthy of attention. How can the dose of polymyxin B be optimized to improve the curative effect and reduce potential nephrotoxicity in elderly patients? There is a lack of relevant research on these questions both domestically and internationally. With the global aging population, these are practical issues that we urgently need to understand to influence treatment decisions. This study retrospectively analyzed the effectiveness and nephrotoxicity of polymyxin B in Chinese elderly patients with CRO infection and analyzed the incidence and related risk factors for polymyxin B-related AKI to provide clinical insights for the selection of polymyxin B therapy in elderly CRO-infected patients.

## Methods

### Study subjects

Patients who received injections of polymyxin B sulfate (≥ 3 days) from any of the clinical divisions of 3 branches of the First Affiliated Hospital of Zhejiang Chinese Medical University from July 2018 to August 2022 were enrolled in the present study. All of them were elderly sepsis patients and the targeted or empirical treatment of CRO infection were considered.

#### The inclusion criteria were as follows

Patients who met the sepsis-3 diagnostic criteria, were aged ≥ 65 years, and were determined to have CRO infection by etiological examination or clinically suspected to have CRO infection.

#### The exclusion criteria were as follows

Those who died within 3 days or 48 h after using polymyxin B.

### Ethics

This study accords with the standards of medical ethics and has been reviewed and approved by the medical ethics committee of the First Affiliated Hospital of Zhejiang Chinese Medical University.

### For the observation indices, the following were collected

The general situation, previous diseases, infection sites, bacterial culture and drug sensitivity test results (type and course of antibiotics), complications, acute physiology and chronic health status score II (APACHE II), serum creatinine (Scr), creatinine clearance, biochemical indices, whether to use vasoactive drugs, polymyxin B daily dose, duration of polymyxin B, microbial evaluation, efficacy evaluation, safety evaluation, 28-d mortality, whether there is AKI, AKI stage, initial onset time of AKI, AKI duration, whether renal replacement therapy is needed, disease outcome, etc.

### Definitions

#### The efficacy evaluation criteria were as follows

① Cure: the patient’s infection is controlled, and all inflammatory indices have returned to normal; ② improvement: the patient’s inflammatory indices have decreased, the body temperature has decreased, and improvement in the clinical manifestations such as decreased ventilator parameters and improved vital signs; ③ ineffective: the patient has had no improvement, including inflammatory indices and clinical manifestations. Curing and improvement are effective treatments.

#### Definition of microbiological evaluation

Patient samples (sputum, blood, abdominal drainage fluid, urine, etc.) were collected and sent to the microbiological laboratory of the First Affiliated Hospital of Zhejiang Chinese Medical University for semiquantitative bacterial culture. If the two results of is greater than or equal to 3 +, it is considered positive. The microbial results were evaluated by repeated microbial culture at the end of treatment. According to the results of semiquantitative microbial culture, ① removal: no protopathogenic bacteria were found in the bacterial culture of the infected site after administration; ② not cleared: no reduction in bacterial count or reduction in bacterial count but not completely cleared.

Sensitivity (S ≤ 2 mg/L) and drug resistance (R ≥ 4 mg/L) were used as clinical turning points for polymyxin B according to the consensus of Chinese experts on drug sensitivity testing and clinical interpretation of polymyxin [11].

#### Renal function assessment

Patients with GFR < 60 mL/min at baseline before infection are defined as having chronic renal insufficiency. Diagnosis of polymyxin B-related AKI: after using polymyxin B, serum creatinine had increased by more than 50% from baseline. AKI is divided into stages I, II and III according to the guidelines of the global organization for the improvement of renal disease prognosis (KDIGO) [12]. Recovery of renal function was defined as the recovery of creatinine to normal levels.

### Statistical analysis

SPSS 26.0 software was used for data analysis. Normally distributed measurement data are expressed as the mean ± standard deviation (x ± s), and a t test was used for comparisons between the groups. Counting data were analyzed using the Chi-square test or Fisher’s exact test. The statistically significant factors and important clinical factors in the univariate analysis were included in the logistic regression model. The independent risk factors for AKI were analyzed by multivariate logistic regression, and the goodness of fit of the logistic regression model was determined by the Hosmer‒Lemeshow test. The receiver operating characteristic curve (ROC) was drawn to evaluate the predictive value of each index for AKI. The survival curves of AKI and non-AKI patients were drawn. P < 0.05 was considered statistically significant.

## Results

### Analysis of the incidence and recovery rate of AKI in elderly patients with CRO treated with polymyxin B

A total of 232 elderly patients with sepsis from CRO infection were analyzed retrospectively. The use of polymyxin B was for ≥ 3 days, and 17 patients with shedding were treated with polymyxin B for less than 3 days. Therefore, a total of 215 elderly patients with sepsis treated with intravenous polymyxin B due to CRO infection were included in this study. The mean age of all patients was 76.03 ± 7.88 years (range 65–100 years), 107 patients (49.8%) were > 75 y, and the APACHE II score was 19.34 ± 6.28. The total all-cause mortality and effective rate were 57.7% (124/215) and 50.7% (109/215), respectively, and the total bacterial clearance rate was 44.2% (95/215). The total incidence of AKI in elderly CRO patients treated with polymyxin B was 37.2% (80/215), and the recovery rate of AKI was 35% (28/80) (Table 1).

**Table 1:** Univariate analysis of factors associated with acute kidney injury (AKI) during Polymyxin B in the treatment with CRO infections

Group	Total [n (%)]	AKI occur [n (%)]	P-value	AKI recovery [n (%)]	P-value
Age (y)					
65–75 y	108 (50.2)	35 (32.4)	0.143	15 (42.9)	0.194
> 75 y	107 (49.8)	45 (42.1)		13 (28.9)	
Sex					
Male	148 (68.8)	57 (38.5)	0.557	17 (29.8)	0.127
Female	67 (31.2)	23 (34.3)		11 (47.8)	
APACHEII score					
< 10	35 (16.3)	5 (14.3)	0.001	3 (60.0)	0.059
10–20	104 (48.4)	36 (34.6)		16 (44.4)	
> 20	76 (35.3)	39 (51.3)		9 (23.1)	
Norepinephrine					
No	63 (29.3)	16 (25.4)	0.006	10 (62.5)	0.019
≤ 0.1 μg/kg/min	98 (45.6)	35 (35.7)		12 (34.3)	
> 0.1 μg/kg/min	54 (25.1)	29 (53.7)		6 (20.7)	
Infection types					
Pneumonia	93 (43.2)	35 (37.6)	0.907	12 (34.3)	1.000
BSIs	49 (22.8)	20 (40.8)		7 (35)	
Intestinal infection	38 (17.7)	13 (34.2)		5 (38.5)	
Other	35 (16.3)	12 (34.3)		4 (33.3)	
Responsible pathogens					
* K.P*	75 (34.9)	32 (42.7)	0.792	10 (31.3)	0.971
* P.A*	57 (26.5)	20 (35.1)		7 (35.0)	
* A.B*	23 (10.7)	7 (30.4)		3 (42.9)	
* E.coli*	24 (11.2)	8 (33.3)		3 (37.5)	
Other	36 (16.7)	13 (36.1)		5 (38.5)	
Polymyxin B daily dose					
1.5 mg/kg/d	65 (30.2)	17 (26.2)	0.028	5 (29.4)	0.03
2.0 mg/kg/d	96 (44.7)	36 (37.5)		18 (50.0)	
2.5 mg/kg/d	54 (25.1)	27 (50.0)		5 (18.5)	
Initial treatment					
Yes	94 (43.7)	32 (34.0)	0.397	16 (50.0)	0.022
No	121 (56.3)	48 (39.7)		12 (25.0)	
Polymyxin B duration (d)					
3–7 d	64 (29.8)	13 (20.3)	0.001	5 (38.5)	0.045
8–14 d	73 (33.9)	27 (37.0)		14 (51.9)	
> 14 d	78 (36.3)	40 (51.3)		9 (22.5)	
Polymyxin B monotherapy					
Yes	31 (14.4)	10 (32.3)	0.538	4 (40.0)	0.725
No	184 (85.6)	70 (38.0)		24 (34.3)	
Polymyxin B combination					
Combined Carbapenems	40 (21.7)	16 (40.0)	0.925	6 (25.0)	0.872
Combined Tigecycline	53 (28.8)	20 (37.7)		8 (40.0)	
Tigecycline + fosfomycin	34 (18.5)	12 (35.3)		4 (33.3)	
Carbapenems + tigecycline	22 (12.0)	10 (45.4)		3 (30.0)	
Other	35 (19.0)	12 (34.3)		5 (41.7)	
Microbiological eradication					
Yes	95 (44.2)	25 (26.3)	0.003	10 (40.0)	0.527
No	120 (55.8)	55 (45.8)		18 (32.7)	
Clinical outcome					
Cure	109 (50.7)	22 (20.2)	< 0.001	12 (54.5)	0.024
Failure	106 (49.3)	58 (54.7)		16 (27.6)	
Prognosis					
Survival	91 (42.3)	15 (18.8)	0.004	9 (60.0)	0.024
Death	124 (57.7)	65 (52.4)		19 (29.2)	

APACHE II: acute physiology and chronic health evaluation II; A.B.: *Acinetobacter baumannii*; COPD: chronic obstructive pulmonary disease, *E. coli*: *Escherichia coli*, *K.P.*: *Klebsiella pneumonia*, *P.A.*: *Pseudomonas aeruginosa*

There were significant differences in the incidence of AKI among the different APACHE II score groups, different doses of norepinephrine groups, different doses of polymyxin B groups and treatment groups (*P* < 0.05). The higher the APACHEII score, the higher the dose of norepinephrine, the higher the dose of polymyxin B and the longer course of treatment resulted in a higher incidence of AKI. The incidence of AKI in the polymyxin B noninitial treatment group, treatment failure group, bacterial nonclearance group and death group was significantly higher than that in the polymyxin B initial treatment group, treatment effective group, bacterial clearance group and survival group (P < 0.05). There was no significant difference among the other groups (*P* > 0.05) (Table 1).

There were significant differences in the recovery rate of AKI among the patients with different doses who were included in the norepinephrine group, polymyxin B group and treatment group (*P* < 0.05). The recovery rate of AKI was higher in the low-dose norepinephrine group, polymyxin B dose 2.0 mg/kg/d group and treatment duration 8–14 d group. The recovery rates of AKI in the polymyxin B initial treatment group, effective treatment group and survival group were significantly higher than those in the polymyxin B noninitial treatment group, ineffective treatment group and death group (*P* < 0.05). There was no significant difference among the other groups (*P* > 0.05). Polymyxin B has a good clinical treatment response, reduces 28-d all-cause mortality, reduces the incidence of nephrotoxicity and improves the recovery rate of AKI (Table 1).

The 28-d all-cause mortality of the AKI group was 81.25% (65/80) and for that of the non-AKI group, it was 43.7% (59/135). The 28-d all-cause mortality of the AKI group was significantly higher than that of the non-AKI group (*P* = 0.024) (Table 1).

### Comparison of different stages of AKI

The total incidence of AKI in elderly CRO patients treated with polymyxin B was 37.2% (80/215), including incidences of 15.81% (34/215) for AKI stage I, 13.95% (30/215) for AKI stage II and 7.44% (16/215) for AKI stage III. For the constituent ratio of AKI patients at each stage, there were significant differences in the following subgroups: between the different doses of norepinephrine group, the different courses of the polymyxin B group, the effective and ineffective treatment group, and the bacterial clearance group and nonclearance group (*P* < 0.05). The constituent ratios of the patients with AKI stage III in the ineffective polymyxin B treatment group and the bacteria not cleared group were higher than those in the effective polymyxin B treatment group and the bacteria cleared group, respectively. There was no significant difference among the other groups (*P* > 0.05). The recovery rate of renal function in different stages of AKI was also different. The recovery rate of renal function in AKI stage I patients was significantly higher than that in AKI stage III patients (*P* < 0.05) (Table 2).

**Table 2:** Comparison of different stages of AKI in each group

Group	Total AKI occur [n (%)]	AKI stage I occur [n (%)]	AKI stage II occur [n (%)]	AKI stage III occur [n (%)]	P-value
Age (y)					
65–75 y	35 (32.4)	16 (45.7)	13 (37.1)	6 (17.1)	0.881
> 75 y	45 (42.1)	18 (40.0)	17 (37.8)	10 (22.2)	
Sex					
Male	57 (38.5)	27 (47.4)	19 (33.3)	11 (19.3)	0.955
Female	23 (34.3)	11 (47.8)	7 (30.4)	5 (21.7)	
APACHEII score					
< 10	5 (14.3)	3 (60.0)	1 (20.0)	1 (20.0)	0.157
10–20	36 (34.6)	20 (55.6)	11 (30.6)	5 (13.9)	
> 20	39 (51.3)	11 (28.2)	18 (46.2)	10 (25.6)	
Norepinephrine					
No	16 (25.4)	11 (68.8)	3 (18.8)	2 (12.5)	0.028
≤ 0.1 μg/kg/min	35 (35.7)	18 (51.4)	12 (34.3)	5 (14.3)	
> 0.1 μg/kg/min	29 (53.7)	7 (22.6)	13 (48.4)	9 (29.0)	
Infection types					
Pneumonia	35 (37.6)	16 (45.7)	14 (40.0)	5 (14.3)	0.545
BSIs	20 (40.8)	5 (25.0)	9 (45.0)	6 (30.0)	
Intestinal infection	13 (34.2)	6 (46.2)	4 (30.8)	3 (23.1)	
Other	12 (34.3)	7 (58.3)	3 (25.0)	2 (16.7)	
Responsible pathogens					
* K.P*	32 (42.7)	11 (34.4)	13 (40.6)	8 (25.0)	0.971
* P.A*	20 (35.1)	10 (50.0)	6 (30.0)	4 (20.0)	
* A.B*	7 (30.4)	3 (42.9)	3 (42.9)	1 (14.3)	
* E.coli*	8 (33.3)	4 (50.0)	3 (37.5)	1 (12.5)	
Other	13 (36.1)	6 (46.2)	5 (38.5)	2 (15.4)	
Polymyxin B daily dose					
1.5 mg/kg/d	17 (26.2)	10 (58.8)	5 (29.4)	2 (11.8)	0.385
2.0 mg/kg/d	36 (37.5)	26 (56.5)	13 (28.3)	7 (15.2)	
2.5 mg/kg/d	27 (50.0)	9 (34.6)	10 (38.5)	7 (26.9)	
Initial treatment					
Yes	32 (34.0)	13 (40.6)	13 (40.6)	6 (18.8)	0.894
No	48 (39.7)	21 (43.8)	17 (35.4)	10 (20.8)	
Polymyxin B Duration (d)					
3–7 d	13 (20.3)	9 (76.9)	2 (15.4)	2 (15.4)	0.027
8–14 d	27 (37.0)	15 (55.6)	8 (29.6)	4 (14.8)	
> 14 d	40 (51.3)	10 (25.0)	20 (50.0)	10 (25.0)	
Polymyxin B monotherapy					
Yes	10 (32.3)	6 (60.0)	3 (30.0)	1 (10.0)	0.457
No	70 (38.0)	28 (40.0)	27 (38.6)	15 (21.4)	
Polymyxin B combination					
Combined Carbapenems	16 (40.0)	5 (31.3)	7 (43.8)	4 (25.0)	0.889
Combined Tigecycline	20 (37.7)	7 (35.0)	9 (45.0)	4 (20.0)	
Tigecycline + fosfomycin	12 (35.3)	5 (41.7)	4 (33.3)	3 (25.0)	
Carbapenems + tigecycline	10 (45.4)	4 (40.0)	3 (30.0)	3 (30.0)	
Other	12 (34.3)	7 (58.3)	4 (33.3)	1 (8.30)	
Microbiological eradication					
Yes	25 (26.3)	16 (64.0)	6 (24.0)	3 (12.0)	0.032
No	55 (45.8)	18 (32.7)	24 (43.6)	13 (23.6)	
Clinical outcome					
Cure	22 (20.2)	15 (68.2)	5 (22.7)	2 (9.1)	0.018
Failure	58 (54.7)	19 (32.8)	25 (43.1)	14 (24.1)	
Prognosis					
Survival	15 (18.8)	9 (60.0)	4 (26.7)	2 (13.3)	0.328
Death	65 (52.4)	25 (38.5)	26 (40.0)	14 (21.5)	
AKI recovery					
Yes	28 (35.0)	16 (47.1)	10 (33.3)	2 (12.5)	0.035
No	52 (65.0)	18 (52.9)	20 (66.7)	14 (87.5)	

APACHE II: acute physiology and chronic health evaluation II; COPD: chronic obstructive pulmonary disease; *A.B.*: *Acinetobacter baumannii; E. coli: Escherichia coli, K.P.: Klebsiella pneumonia; P.A.: Pseudomonas aeruginosa*

### Occurrence of renal injury in the different groups

AKI occurred in the effective treatment group, 22 cases were considered to be caused by polymyxin B nephrotoxicity, the total number of cases was 215, and the incidence rate was 10.2% (22/215). However, acute renal injury occurred in the ineffective treatment group, which was first considered to be related to the aggravation of infection, but the nephrotoxicity of polymyxin B could not be completely excluded. Therefore, the incidence of nephrotoxicity of polymyxin B was in the range of 10.2–37.2% (22/215–80/215) (Table 3).

**Table 3:** The occurrence and improvement of renal damage in different groups

Group	No-AKI incidence [n (%)]			AKI incidence [n (%)]		
	[n (%)]	Effective group	Ineffective group	[n (%)]	Effective group	Ineffective group
No renal injury group (n = 124)	96 (77.4%)^a^	62 (64.6%)	34 (35.4%)	28 (22.6%)	5 (17.9%)	23 (82.1%)
Chronic renal injury (n = 48)	13 (27.1%)	8 (61.5%)	5 (38.5%)	35 (72.9%)	12 (34.3%)	23 (65.7%)
Acute renal injury group (n = 43)	26 (60.5%)^b^	17 (65.4%)	9 (34.6%)	17 (39.5%)	5 (29.4%)	12 (70.6%)
Total (n = 215)	135 (62.8%)	87 (64.4%)	48 (35.6%)	80 (37.2%)	22 (27.5%)^c^	58 (72.5%)

Comparison of no AKI incidence: compared with the chronic renal injury group before treatment, ^a^P < 0.01; Acute renal injury group before treatment versus the chronic renal injury group before treatment, ^b^P < 0.01

Comparing AKI incidence, ^c^P < 0.05 in the effective treatment group versus the ineffective treatment group

When comparing the no AKI incidence, the incidences in the group without renal damage before treatment and the group with acute renal injury before treatment were significantly higher than that in the group with chronic renal damage before treatment (*P* < 0.01). The incidence in the group without renal damage before treatment was higher than that in the group with acute renal injury before treatment, but the difference was not significant (P > 0.05). When comparing the AKI incidence, the incidence in the effective group was significantly lower than that in the ineffective group (*P* < 0.05) (Table 3).

### Univariate and multivariate analysis of AKI in elderly CRO patients treated with polymyxin B

The univariate regression analysis shows that the predictive factors of AKI in elderly CRO patients treated with polymyxin B are APACHE II score, chronic renal insufficiency, high dose of polymyxin B, long duration of polymyxin B, high dose of norepinephrine, effective bacterial clearance and treatment. The above multivariate regression analysis shows that the predictors of AKI in elderly CRO patients treated with polymyxin B are high APACHE II score, long duration of polymyxin B, chronic renal insufficiency and effective treatment (Table 4).

**Table 4:** Univariate and multivariate logistic regression analysis of AKI in elderly CRO patients treated with polymyxin B

Characteristic	Univariate analysis		Multivariate analysis	
	Odds ratio (95% confidence interval)	P-value	Odds ratio (95% confidence interval)	P-value
APACHE II score	0.944 (0.897, 0.993)	0.027	2.084 (1.205, 3.604)	0.009
Chronic kidney disease	7.299 (3.544, 15.034)	0.000	6.705 (2.883, 15.594)	0.000
Polymyxin B high dose	1.680 (1.144–2.466)	0.008		
Duration of polymyxin B treatment (days)	1.870 (1.302, 2.686)	0.001	1.651 (1.029, 2.649)	0.037
Norepinephrine dose	1.853 (1.252, 2.743)	0.002		
Microbiological without eradication	2.390 (1.325, 4.235)	0.004	3.573 (1.779, 7.719)	0.000
Clinical failure	4.778 (2.612, 8.743)	0.000		

### The predictors of AKI in elderly CRO-infected patients after polymyxin B use were as follows

**Fig. 1:**
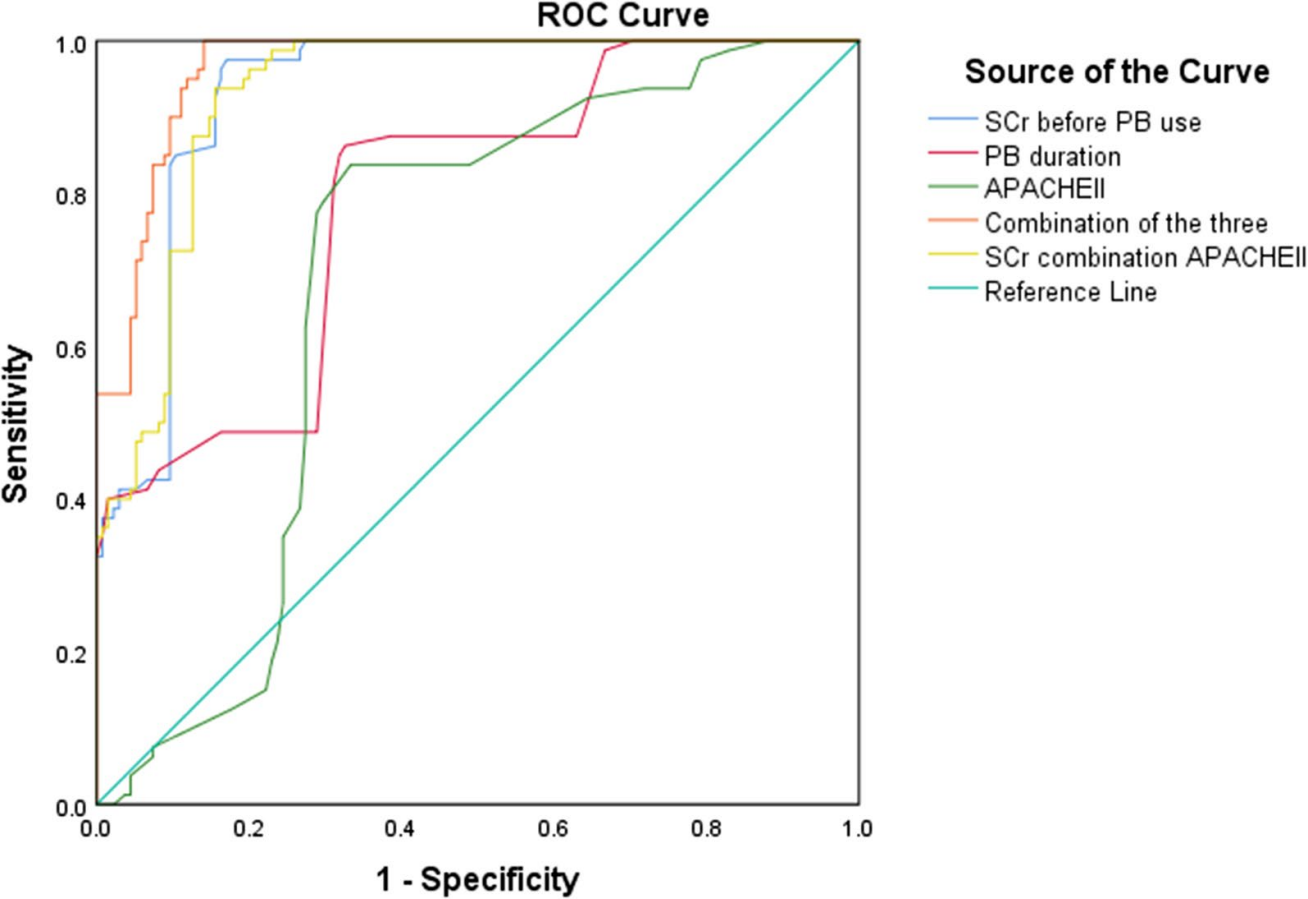
The ROC curves for predicting AKI in elderly CRO infected patients on serum creatinine, APACHE II score before polymyxin B treatment and polymyxin B duration. ROC, receiver operating characteristic; AUC, area under the curve; PB, polymyxin B; Scr, serum creatinine; AKI: acute kidney injury; CRO, carbapenem resistant organism

Creatinine, APACHE II score before polymyxin treatment and polymyxin use duration. The ROC curve showed that the cutoff values for predicting AKI were 73 mmol/L of serum creatinine before polymyxin B use, 10.5 days of PB duration, and APACHE II score of 14.5. (Fig. 1 and Table 5).

**Table 5:** The value of APACHEII, SCr before PB use, PB duration and their combination in predicting AKI in elderly CRO infected patients

Indicator	AUC	Youden index	Cut-off	Sensitivity (%)	Specificity (%)
APACHEII	0.693	0.504	14.5	83.8	66.7
SCr before PB use	0.931	0.805	73	97.5	83
PB duration	0.792	0.573	10.5	86.5	67.4
Combination of the three	0.965	0.859	0.228	100	85.9
SCr combination APACHEII	0.930	0.782	0.357	93.8	84.4

PB, polymyxin B; Scr, serum creatinine; AKI: acute kidney injury; CRO, carbapenem resistant organism; AUC, area under the curve

### The survival curve between the AKI group and the non-AIK group

**Fig. 2:**
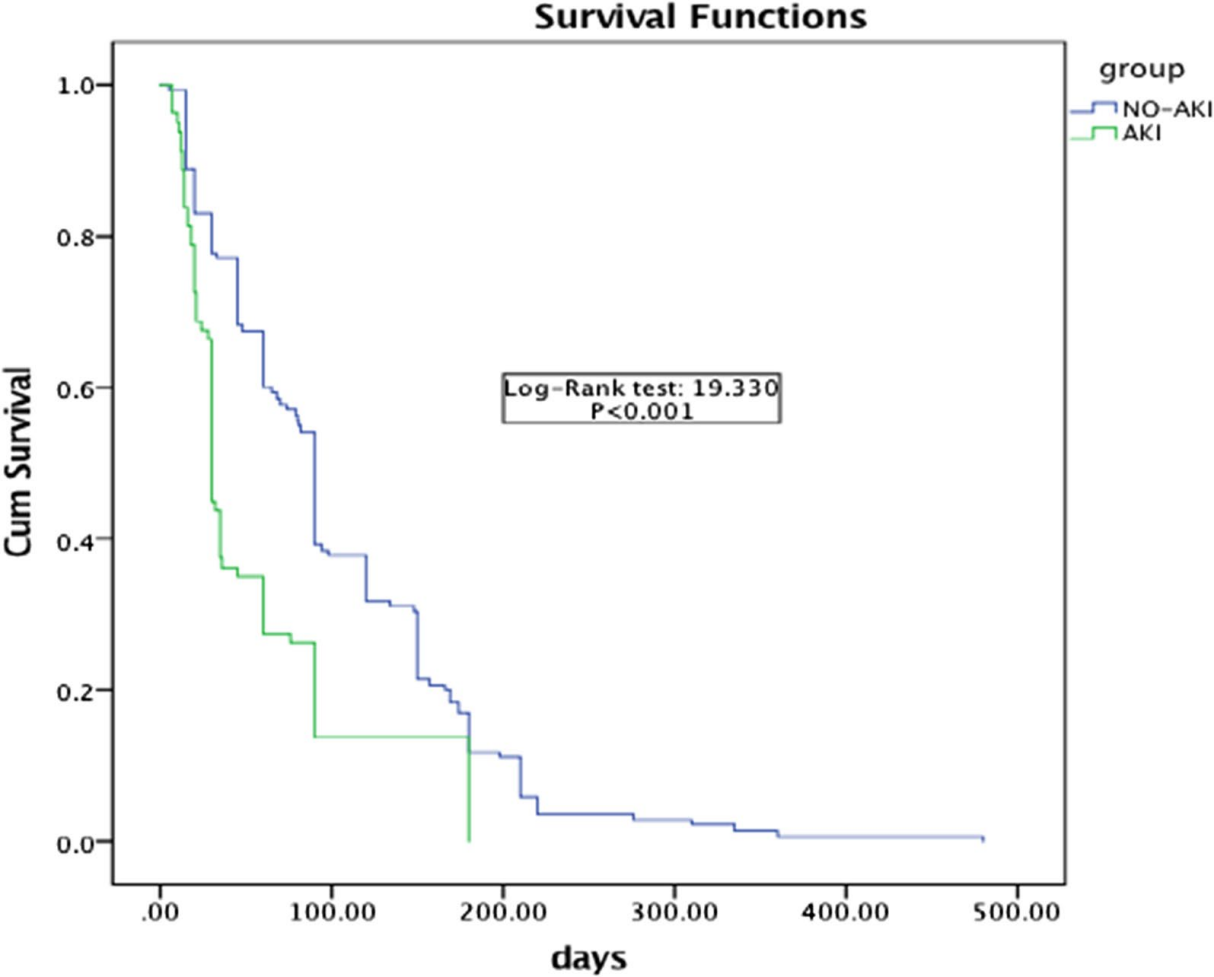
Survival curve of AKI group and non AIK group. AKI: acute kidney injury

The survival time of the non-AKI group was significantly longer than that of the AKI group (*P* < 0.001) (Fig. 2).

## Discussion

Studies have discussed the efficacy and safety of polymyxin B in European and American patients [13, 14], but the efficacy and nephrotoxicity of polymyxin B in elderly infected patients have not been reported. A total of 215 cases of sepsis caused by CRO infection in the elderly were analyzed retrospectively. The total effective rate was 50.7% (109/215), and the total bacterial clearance rate was 44.2% (95/215). The overall incidence of AKI in this study was 37.2% (80/215), the recovery rate of AKI was 35% (28/80), and the incidence of polymyxin B-related AKI ranged from 10.2% to 37.2% (22/215–80/215). Gomes EC et al. [15] found that 31 (33%) of 94 patients with polymyxin B-related AKI in Brazil recovered renal function within 1 year, which is similar to the results of this study. Although our subjects were elderly patients, our study and the study in Brazil have shown that intravenous polymyxin B can improve bacterial clearance and clinical efficiency in infected patients. The clearance of polymyxin B does not depend on glomerular filtration, but the mechanism of its nephrotoxicity is to increase the permeability of the renal tubular epithelial cell membrane, resulting in the inflow of cations, anions and water, resulting in cell swelling and cell lysis [16]. Mohammad et al. [17] detected that the concentration of polymyxin B in human renal tubular cells is approximately 4760 times higher than the extracellular concentration, indicating that the reabsorption of polymyxin B in renal tubules leads to abnormal accumulation in cells and direct damage to tubular cells. This process may explain how polymyxin B causes damage to the kidney when it is cleared as a nonrenal drug. Previous studies reported that the incidence of polymyxin B-induced nephrotoxicity ranged from 18.2% to 46.1% [18, 19]. However, different definitions of nephrotoxicity were applied, and a broad polymyxin B dose was administered, which may explain the different results in previous studies. The incidence of polymyxin B-related nephrotoxicity reported by Kady et al. [20] is lower than that observed by us (23.1% and 37.2%), which is related to their use of the RIFLE standard to define AKI, while we used the KDIGO standard. The subjects were elderly patients who were more prone to renal injury caused by infection and drug induction, which may explain the high incidence of nephrotoxicity.

Infection can cause acute renal injury in patients but can be reversed by appropriate antibiotic treatment [21]. Studies have shown that patients with existing renal dysfunction before polymyxin B treatment may be at higher risk for polymyxin B nephrotoxicity, although it is unclear whether this renal dysfunction is caused by a baseline or severe infection [22]. In this study, 22 cases of AKI occurred in the effective treatment group, which was considered to be caused by polymyxin B nephrotoxicity. The total number of cases was 215, and the AKI incidence was 10.2% (22/215) in the effective treatment group. However, in the ineffective treatment group, acute renal injury was first considered to be related to the aggravation of infection, but the nephrotoxicity of polymyxin B could not be completely excluded. Therefore, the nephrotoxicity of polymyxin B in this study was in the range of 10.2–37.2%. Without sufficient anti-infective treatment and good anti-infective effects, it may be difficult to distinguish polymyxin B-related AKI from infection-induced AKI. For the incidence of AKI, the higher the APACHE II score, the greater the norepinephrine dose, the higher the polymyxin B dose and the longer the duration of PB treatment resulted in a higher incidence of AKI. The incidence of AKI in the polymyxin B noninitial treatment group, treatment ineffective group, bacteria nonclearance group and death group was significantly higher than that in the polymyxin B initial treatment group, treatment effective group, bacteria clearance group and survival group (*P* < 0.05). The recovery rate of AKI in the effective treatment group was higher than that in the ineffective group, indicating that the more serious the condition of elderly patients was, the more likely they were to have AKI with high-dose and long-term polymyxin B. The high bacterial clearance, effective treatment group and initial treatment group had good infection control, which could prevent AKI caused by infection. In this study, why was there renal function improvement in the treatment ineffective group? It may be that renal perfusion is guaranteed or that other causes of renal damage were corrected. Compared with the group with acute renal injury before PB treatment, the no-AKI incidence in the group with no renal damage before PB treatment was significantly higher than that of chronic renal damage before PB treatment (*P* < 0.01); when comparing the AKI incidence, the effective group was significantly lower than the ineffective group (*P* < 0.05). It showed that effective treatment was very important, which not only reduced the 28-d all-cause mortality and the incidence of AKI but also improved the recovery rate of AKI. Compared with the acute renal damage group before PB treatment, acute renal injury was more likely to be aggravated on the basis of previous chronic renal damage because simple acute renal damage can restore renal function through anti-infection treatment or ensuring renal perfusion, but chronic renal damage is difficult to correct. When polymyxin B must be used, it may make things worse and cause damage to the kidney again. To ensure the anti-infective effect and renal function, if the deterioration of renal function cannot be reversed by anti-infective treatment after polymyxin B treatment, CRRT treatment can be used to improve the recovery rate of AKI and improve the prognosis.

The survival curves of the AKI group and non-AKI group showed that the survival time of the non-AKI group was significantly longer than that of the AKI group (*P* < 0.001), and the mortality of patients without AKI was 37.55% lower than that of AKI, indicating that patients without polymyxin B-related AKI had a better prognosis, long survival time and low mortality. Multicenter studies showed that the mortality of patients with polymyxin B-related AKI was 17% higher than that of patients without AKI [23], and our study showed that the mortality of patients with AKI was 37.55% higher than that of patients without AKI, which was significantly higher than the above research results. This is related to the fact that our selected subjects were elderly patients. Once AKI occurs, the prognosis is extremely poor. For the AKI recovery rate, Gomes’s research shows that the recovery rate of renal function in patients with polymyxin B-related AKI is 33% [15]; most AKI caused by polymyxin is reversible, in part because of the high reabsorption rate of the kidney, especially the proximal renal tubular cells in the renal cortex; and this is due to the saturable active form of Polymyxin B [24]. Our subgroup analysis showed that the recovery rate of stage I AKI was significantly higher than that of stage III AKI (47.1% vs. 12.5%). The higher the norepinephrine dose group, polymyxin B dose 2.0 mg/kg/d group and treatment duration 8–14 d group, the higher the recovery rate of AKI. The recovery rates of AKI in the polymyxin B initial treatment group, effective treatment group and survival group were significantly higher than those in the polymyxin B noninitial treatment group, ineffective treatment group and death group (*P* < 0.05). Appropriate duration and dose of polymyxin B, initial treatment and effective treatment can improve the recovery rate of AKI. This suggests that once polymyxin B-related AKI occurs in elderly patients with infection, it is necessary to actively treat the primary disease to improve the prognosis of patients. It may also be of certain significance to improve the prognosis by correcting possible reversible risk factors, avoiding aggravation of AKI, and protecting or reversing renal function to the greatest extent.

It is of great significance to analyze the risk factors for polymyxin B-related AKI. Patients with many high-risk factors can avoid the occurrence of AKI to a certain extent by avoiding polymyxin B. A retrospective analysis revealed that the daily dose of polymyxin B, the use of vasoactive drugs and the length of stay in the ICU were independent risk factors for polymyxin B-related AKI in ICU patients [25]. A meta-analysis showed that advanced age, high daily dose, underlying diseases such as diabetes, and associated nephrotoxic drugs were independent predictors of nephrotoxicity [26]. Animal experiments have shown that the pathogenesis of polymyxin B-related AKI is the reabsorption and deposition of polymyxin in renal proximal convoluted tubular cells; this then induces oxidative stress, cell cycle arrest and apoptosis [27, 28]. This cytotoxic damage is dose dependent. The PK study of XuBen et al. showed that different renal functions have different effects on the clearance rate of polymyxin B. Monte Carlo simulation was also carried out to evaluate the possibility of using lower doses of polymyxin B to achieve optimal exposure and reduce toxicity in patients with renal insufficiency [29]. Univariate analysis showed that high APACHE II score, chronic renal insufficiency, high dose and long duration of polymyxin B, high dose of norepinephrine, nonclearance of bacteria and ineffective treatment were predictors of AKI in elderly CRO patients treated with polymyxin B. The multivariate regression analysis that included the above factors showed that high APACHE II score, long duration of polymyxin B, chronic renal insufficiency and ineffective treatment were predictors of AKI in elderly CRO patients treated with polymyxin B. Further ROC curve analysis showed that the cutoff value of serum creatinine before polymyxin B was 73 mmol/L; that is, the sensitivity and specificity of predicting AKI in elderly CRO patients with a cutoff greater than 73 mmol/L were 97.5% and 83%, and the AUC was 0.931. When polymyxin B was used for more than 10.5 days, the sensitivity and specificity of predicting AKI were 86.3% and 67.4%, and the AUC was 0.792. The APACHE II score was greater than 14.5; the sensitivity and specificity of predicting AKI were 83.8% and 66.7%; and the AUC was 0.693. The above results do not mean that AKI will occur when the levels of polymyxin B are higher than the cutoff value, but the probability of occurrence is higher than that lower than the cutoff value. The above cutoff values are lower than we expected, which may be related to the serious condition and more complications of the included elderly patients, such as more hematology and severe patients, low immunity and easy damage to renal function. We can further analyze the cutoff values of different subgroups. Of course, we do not recommend that the dose of polymyxin B be reduced in elderly patients with renal dysfunction because treatment failure may increase the incidence rate and mortality risk. This study and Rigatto's multicenter prospective study confirm this [19].

The above results suggest that for elderly patients with severe disease, basic renal insufficiency, shock, and treatment with high-dose and long-term polymyxin B, clinicians should be particularly alert to the occurrence of AKI, early correction of shock, reduction of drug dose and use time. At present, the drug dose selection of polymyxin B is mostly calculated according to the actual weight of patients. For elderly patients with great changes in pathophysiological state and drug metabolism, the plasma protein binding rate of polymyxin B in general patients and severe patients is very different (50–70% vs. more than 90%) [22]. The above rough calculation method has difficulty meeting the needs of higher levels. Therefore, the consensus of Chinese experts on the clinical application of polymyxin recommends titrating drug dose adjustments with therapeutic drug monitoring (TDM) [30]. However, most TDM detection methods detect free + bound drugs in blood samples, which cannot directly reflect the concentration of free drugs that can exert efficacy in severe patients. More pharmacokinetic studies are urgently needed to meet the clinical needs, such as Target Concentration Intervention (TCI) [31]. The current Polymyxin B PK/PD parameters have poor operability in practical work. There is an urgent need for convenient and simple evaluation index standards to seek a more accurate dose‒response relationship between polymyxin B drug dose and AKI to reduce the occurrence of drug-related AKI.

This study was a multicenter retrospective study. In the future, a well-designed multicenter, prospective controlled trial is needed to study the effectiveness of polymyxin B in elderly patients with infection, its effect on renal function and the treatment of preventing renal damage. In this study, the weight of most critically ill patients was difficult to obtain accurately, and the dosage of polymyxin B was relatively rough. In the later stage, the TCI of polymyxin B can be used to guide individualized administration to further understand the relationship between drug dose and AKI in elderly patients.

In conclusion, rational use of polymyxin B is safe and effective in elderly patients with CRO infection. The high bacterial clearance, polymyxin B effective treatment and initial treatment were conducive to infection control, which can prevent AKI caused by infection and reduce the nephrotoxicity of polymyxin B. The appropriate course and dose of polymyxin B, initial treatment and effective outcome can improve the recovery rate of AKI. This provides a certain basis for the treatment of CRO-infected elderly patients with polymyxin B and has clinical guiding significance.

## Data Availability

Not applicable.

## Abbreviations

*CRO*: *Carbapenem**-**resistant* *Organism**s*
*CRE*: *Ca* *rbapenem* *-* *resistant Enterobacteriaceae*
*CRAB*: *Carbapenem-resistant Acinetobacter baumannii*
*CRPA*: *Carbapenem-resistant Pseudomonas aeruginosa*
EUCAST: The European Commission for antimicrobial susceptibility testing
MIC: Minimum inhibitory concentration of antibiotics
APACHE II: Acute physiology and chronic health evaluation II
ICU: Intensive care unit
MODS: Multiple organ dysfunction syndrome
ARDS: Acute respiratory distress syndrome
COPD: Chronic obstructive pulmonary disease
*K.P.*: *Klebsiellapneumonia*
*P**.**A**.*: *Pseudomonas aeruginosa*
*E coli*: *Escherichia coli*
*E.fm*: *Enterococcus* *faecium*
*A.B.*: *Acinetobacter baumannii*
*MDR-AB*: *Multidrug-resistant Acinetobacter baumannii*
*KPC*: *Carbapenemase produced in Klebsiella pneumoniae*
PK/PD: Pharmacokinetic/Pharmacodynamics
KDIGO: Kidney Disease Improving Global Outcomes, or improved global renal disease prognosis Organization
AKI: Acute renal injury
ROC: Receiver operating characteristic curve
GFR: Glomerular filtration rate
Scr: Serum creatinine
AUC: Area under the curve
PB: Polymyxin B
TDM: Therapeutical drug monitoring
TCI: Target concentration intervention
